# Structural Bases of Zoonotic and Zooanthroponotic Transmission of SARS-CoV-2

**DOI:** 10.3390/v14020418

**Published:** 2022-02-17

**Authors:** Emily Clayton, Jacob Ackerley, Marianne Aelmans, Noor Ali, Zoe Ashcroft, Clara Ashton, Robert Barker, Vakare Budryte, Callum Burrows, Shanshan Cai, Alex Callaghan, Jake Carberry, Rebecca Chatwin, Isabella Davies, Chloe Farlow, Samuel Gamblin, Aida Iacobut, Adam Lambe, Francesca Lynch, Diana Mihalache, Amani Mokbel, Santosh Potamsetty, Zara Qadir, Jack Soden, Xiaohan Sun, Alexandru Vasile, Otto Wheeler, Mohammed A. Rohaim, Muhammad Munir

**Affiliations:** Division of Biomedical and Life Sciences, Faculty of Health and Medicine, Lancaster University, Lancaster LA1 4YG, UK; e.clayton3@lancaster.ac.uk (E.C.); j.ackerley@lancaster.ac.uk (J.A.); m.aelmans@lancaster.ac.uk (M.A.); n.a.ali@lancaster.ac.uk (N.A.); z.ashcroft@lancaster.ac.uk (Z.A.); c.ashton3@lancaster.ac.uk (C.A.); r.barker4@lancaster.ac.uk (R.B.); v.budryte@lancaster.ac.uk (V.B.); c.burrows1@lancaster.ac.uk (C.B.); s.cai6@lancaster.ac.uk (S.C.); a.callaghan4@lancaster.ac.uk (A.C.); r.j.carberry@lancaster.ac.uk (J.C.); r.chatwin@lancaster.ac.uk (R.C.); i.davies1@lancaster.ac.uk (I.D.); c.farlow@lancaster.ac.uk (C.F.); s.gamblin@lancaster.ac.uk (S.G.); a.iacobut@lancaster.ac.uk (A.I.); a.lambe@lancaster.ac.uk (A.L.); f.lynch@lancaster.ac.uk (F.L.); d.mihalache@lancaster.ac.uk (D.M.); a.mokbel@lancaster.ac.uk (A.M.); s.potamsetty@lancaster.ac.uk (S.P.); z.qadir@lancaster.ac.uk (Z.Q.); j.soden@lancaster.ac.uk (J.S.); x.sun15@lancaster.ac.uk (X.S.); a.vasile1@lancaster.ac.uk (A.V.); o.wheeler@lancaster.ac.uk (O.W.); m.a.rohaim@lancaster.ac.uk (M.A.R.)

**Keywords:** SARS-CoV-2, zoonotic, transmission, ACE2, eradication

## Abstract

The emergence of multiple variants of severe acute respiratory syndrome coronavirus 2 (SARS-CoV-2) highlights the importance of possible animal-to-human (zoonotic) and human-to-animal (zooanthroponotic) transmission and potential spread within animal species. A range of animal species have been verified for SARS-CoV-2 susceptibility, either in vitro or in vivo. However, the molecular bases of such a broad host spectrum for the SARS-CoV-2 remains elusive. Here, we structurally and genetically analysed the interaction between the spike protein, with a particular focus on receptor binding domains (RBDs), of SARS-CoV-2 and its receptor angiotensin-converting enzyme 2 (ACE2) for all conceivably susceptible groups of animals to gauge the structural bases of the SARS-CoV-2 host spectrum. We describe our findings in the context of existing animal infection-based models to provide a foundation on the possible virus persistence in animals and their implications in the future eradication of COVID-19.

## 1. Introduction

During late 2019, a novel severe acute respiratory syndrome coronavirus 2 (SARS-CoV-2) emerged as the causative agent of the current coronavirus disease 19 (COVID-19) pandemic. SARS-CoV-2 is a member of the *Coronaviridae* family, which is part of the largest group of viruses known as the *Nidovirales* order [1]. So far, seven human coronaviruses (HCoVs), namely HCoV-229E, HCoV-OC43, HCoV-NL63, HCoV-HKU1, severe acute respiratory syndrome coronavirus (SARS-CoV), Middle East respiratory syndrome coronavirus (MERS-CoV), and SARS-CoV-2 have been identified. The HCoVs (HCoV-229E, HCoV-OC43, HCoV-NL63, HCoV-HKU1) cause flu-like symptoms in humans and are responsible for 15–30% of common cold cases worldwide. Occasionally, severe and life-threatening lower respiratory tract infections can occur in infants, elderly people, or immunocompromised patients [2]. On the other hand, other coronaviruses can be much more pathogenic, namely those that have recently emerged via zoonotic spillover events. This occurs when a coronavirus strain in an animal reservoir evolves and mutates into a new human coronavirus that can infect human populations.

SARS-CoV-2 is the most recent zoonotic coronaviruses to cause devastation in humans compared to previous coronavirus outbreaks including SARS and MERS-CoV [3]. The genome of SARS-CoV-2 shares 80% of its identity with the cause of the previous SARS outbreak (SARS-CoV) and interestingly, SARS-CoV-2 has now been identified as having a 96% genome similarity to the bat coronavirus BatCoV (i.e., RaTG13), which is believed to be an ancestral coronavirus. These zoonotic viruses are believed to have originated and emerged from bat coronaviruses and collectively exist as extremely pathogenic, highly transmissible viruses [4]. 

Invasion of a host cell is the initial phase in SARS-CoV-2 infection, which is mediated by the spike (S) glycoprotein. S1 and S2 are two subunits of the S-protein, which is a glycosylated type I membrane protein [5]. The S-protein exists in a trimeric pre-fusion state that is cleaved into two subunits by the host furin protease. The N-terminal S_1_ subunit contains the receptor-binding domain (RBD), which enables binding to the host cell receptor, namely angiotensin converting enzyme 2 (ACE2) [6]. Binding of RBD to ACE2, followed by additional cleavage of the S2 subunit at a second specific site by the host serine protease TMPRSS2, are critical for triggering the cleavage of S1 and S2, resulting in conformational changes in S2 that are responsible for viral and host membrane fusion and virus entry [6,7]. 

Both SARS-CoV-2 and SARS-CoV enter host cells via the interaction with the ACE2 receptor that is expressed on the surface of many pulmonary and extra-pulmonary cell types, including renal, cardiac, intestinal, and endothelial cells [8,9]. Expression of ACE2 is highly conserved across a variety of animals due to its important roles in physiology of the vascular, renal and myocardial systems [10]. Due to ACE2 acting as the central binding site required for SARS-CoV-2 entry, variations in amino acids within the ACE2 sequence, especially in residues essential in binding with the RBD of the S-protein, are likely to have a significant impact on host susceptibility to the virus [10].

The emergence of SARS-CoV-2 variants, particularly those with critical mutations in the RBD of the S-protein fuels the global spread of SARS-CoV-2. Several of these novel variants can spread more easily, particularly those recognized as Variants of Concern (VOCs) by the World Health Organization [11]. In addition, these VOCs have caused reinfections or vaccine breakthrough infections via reducing sensitivity to neutralizing antibodies generated by natural COVID-19 infection or first-generation COVID-19 vaccinations [12,13]. The Delta variant (B.1.617.2) was found for the first time in India in late 2020, and by 24 August 2021, it had reached over 163 countries. In June 2021, the World Health Organization declared the Delta strain as a VOC [14]. Later, a novel SARS-CoV-2 lineage B.1.1.529 (Omicron), first reported by South Africa and Bostwana, was classified by the World Health Organization as a VOC on 26th November 2021 [15]. This is particularly worrisome because of the unusually large number of mutations in the variant, including those that are known to cause escape from neutralizing antibodies and increased binding to the host cell receptor ACE2.The S-protein of Omicron has 30 single point mutations, three deletion mutations, and one insertion mutation when compared to the SARS-CoV-2 wild type (Wuhan ancestral strains). Surprisingly, the Omicron RBD possesses 15 mutations, 10 of which are within the receptor-binding motif (RBM), which directly interacts with human angiotensin-converting enzyme (hACE2) and most monoclonal antibodies (mAbs). In comparison, the currently prevalent variant Delta (B.1.617.2) has just two mutations in its RBM, with additional K417N and E484K changes. It can therefore be determined that the Omicron variant may have a major impact on the binding affinity and efficacy of currently available mAbs [16]. 

Due to a large and diverse number of species possessing ACE2 receptors [17], it is imperative to investigate their possible susceptibility to SARS-CoV-2 infection, as this could have substantial implications in the transmission dynamics and prospective zooanthroponotic cases of COVID-19. These analyses will provide bases for the potential virus persistence in animals and their implications in the future eradication of COVID-19.

## 2. Interaction between SARS-CoV-2 and Host Cell Receptors

For viral attachment and entry, SARS-CoV-2 efficiently uses various host factors in humans. The SARS-CoV-2’s S-protein binds to the hACE2 receptor more effectively than that of closely related coronaviruses [18]. In addition, SARS-CoV-2 employs additional attachment factors such as human heparan sulphate, C-type lectin receptors, DC-SIGN, L-SIGN, and sialic acid-binding Ig-like lectin 1 (SIGLEC1) [19,20]. The endosomal proteases, cathepsins and human transmembrane serine protease 2 (TMPRSS2) are essential for the activation of the S-protein to allow membrane fusion [6]. Upon binding of the S-protein to the ACE2 receptor, a series of signalling events occur, which eventually enable the virus to enter the host cell. The ability of the virus to do this is primarily dependent on its ability to recognise the ACE2 receptor and interact with it efficiently. Variations in the amino acids involved in this interaction may account for the differences observed in susceptibility to the virus, along with differences in symptom severity observed between hosts.

**Figure 1:**
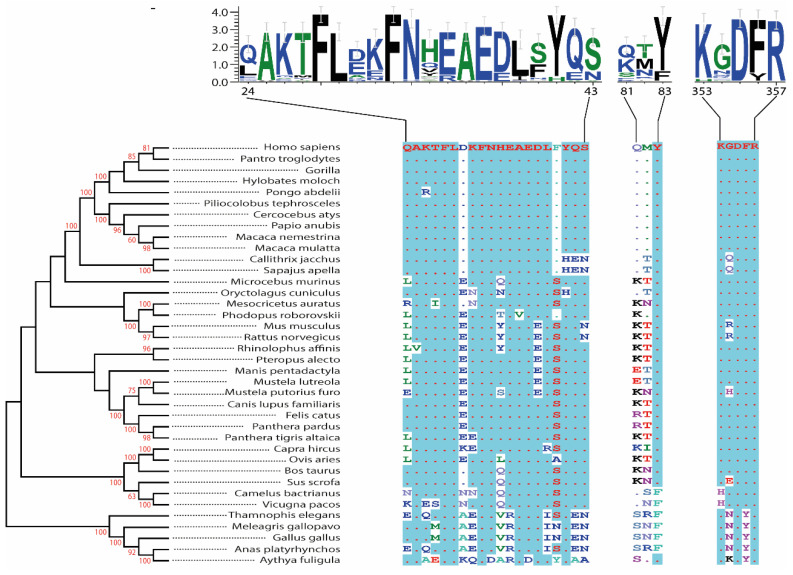
A phylogenetic tree of ACE2 proteins assembled using the neighbour-joining method conducted in MEGA7 with ambiguous positions removed. The tree is drawn to scale, and support was provided with 1000 bootstraps. ACE2 protein sequence alignment and evolutionary relationships of different species. The amino acid residues at critical binding sites for the SARS-CoV-2 spike receptor-binding domain were outlined. The accession numbers of all sequences used in the phylogenetic analysis are: NM001371415, XM016942979, XM019019204, XM032756617, XM024240245, XM023199053, XM012035809, XM021933040, XM011735203, NM001135696, XM008988993, XM032285963, XM020285237, XM002719845, XM005074209, MW075232, NM001130513, NM001012006, MT394225, XM006911647, MT038416, MT560518, NM001310190, NM001165260, NM001039456, XM019417964, XM007090080, NM001290107, XM012106267, NM001024502, NM001123070, XM010968001, XM006212647, XM032227043, XM019612009, XM416822, and XM013094461.

SARS-like coronaviruses rely on the S-protein for cell entry [18]. The genomic sequencing of SARS-CoV-2 determined a similarity of ~80% with SARS-CoV, thus indicating the likelihood of SARS-CoV-2 also utilising S-protein for its cellular entry. Furthermore, when sequencing the S-protein of both coronaviruses, 76% similarity was observed for the whole protein, ~74% for the RBD, and ~52% for the RBM. As viral entry is initiated by the binding of the RBD of the S-protein [8,9,18], it was suggested that as already observed with SARS-CoV, hACE2 receptors may also be susceptible to and provide a prime target for SARS-CoV-2 [8,9,18]. The SARS-CoV-2 S subunit 1 RBD (amino acids 303 to 537) involves the RBM residues responsible for interactions with ACE2s’ outer surface (Figure 1). SARS-CoV-2 could feasibly use the ACE2 receptor to infect a range of non-human and non-bat hosts. To this end, we analysed the hACE2 as well as orthologues from other vertebrate species based on the phylogeny and amino acid identity for the interaction sites, including companion animals (dogs, cats, rabbits, hamsters, rats, and ferrets), livestock species (chickens, camels, cattle, sheep, goats, and pigs), bat species (horse-shoe bat, fruit, and flying fox bat), and two species confirmed or suspected to be associated with coronavirus outbreaks (civet and pangolin) (Figure 1). Our analysis revealed that there is a 62% to 99% sequence identity between these proteins at the amino acid level and 76% to 99% when excluding the chicken sequences, and their phylogenetic relationships are largely consistent with vertebrate phylogeny (Figure 1).

### 2.1. Human

On the verge of the S-ACE2 receptor interface, it appeared that SARS-CoV-2 has acquired advantageous mutations in the RBM, resulting in successful cellular entry and higher transmissibility rates, when compared to SARS-CoV. A total of seven unique residues have been attributed to the natural selection of SARS-CoV-2 for human [21,22]. Out of these seven unique residues, five are responsible for enhanced binding efficacy (Leu455, Phe486, Gln493, Ser494, and Asn501). Residue Asn501 is crucial for S-ACE2 stability due to it interacting with hotspot-353 of the ACE2 receptor. Furthermore, Gln493 interacts with ACE2 hotspot-31, enhancing this stability. Additionally, Leu455 and Phe486 of SARS-CoV-2 RBM provide more favourable interactions with ACE2 hotspot-31 compared to SARS-CoV Tyr442 and Leu472 residues. Overall, these five key residues encourage an enhanced stable binding state in hACE2-S complexes in SARS-CoV-2 [21]. Moreover, Ali and Vijayan, [22] have conducted dynamic-simulations, assessing the significance of such mutations whereby an enhanced SARS-CoV-2-RMB-ACE2 interface was observed. They identified several interactions promoting greater binding stability via increased hydrophobic interactions and higher free-energy binding [22]. These findings collectively support the identification of hACE2 as a susceptible SARS-CoV-2 target that is utilised in cellular entry. It is important to note that multiple factors including age, cell type, species, and genetic polymorphisms might play a role in defining S-ACE2 interactions.

**Figure 2:**
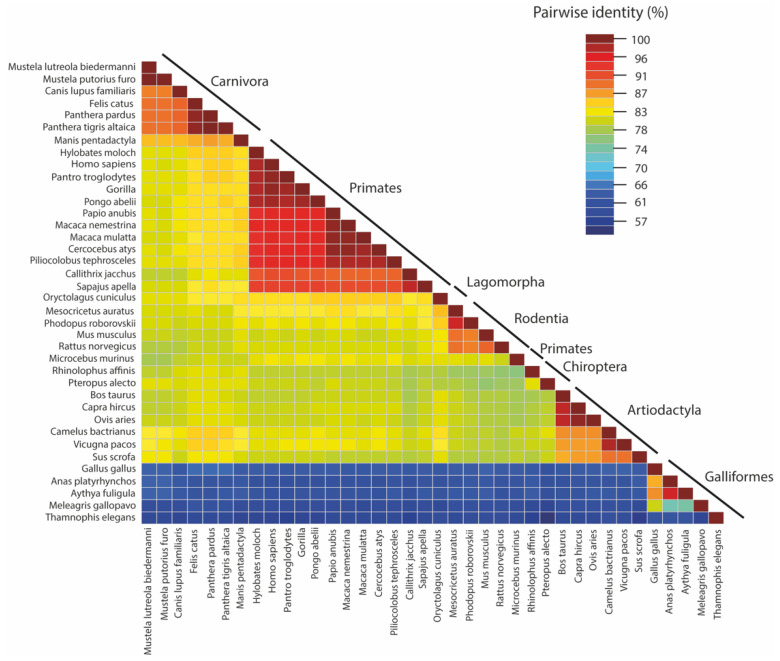
The pairwise identities plot of ACE2 protein sequences aligned by MAFFT and displayed by Sequence Demarcation Tool (SDT) software.

The affinity for the RBM by ACE2 is determined by complimentary charges of the interacting structures. The surface deep channel of the ACE2 receptor and its surrounding ridges are highly negative, containing residues D136, E150, N154, D157, D292, D295, and D299, which have high areas of solvent accessibility (ASA) values. These ridges may provide a possible binding site for the positively charged RBD of the S glycoprotein [23]. Thus far, analysis of the ACE2 locus has identified 2266 polymorphisms [24]. Recently, there has been evidence suggesting that these ACE2 polymorphisms may play a role in susceptibility to SARS-CoV-2. While the ACE2 receptors show a variable degree of divergence between animal classes (Figure 2), structural changes in the ACE2 receptor have also been observed that alter the interaction with the S protein of SARS-CoV-2 [25].

Although the overall spatial structure of the ACE2 varies slightly, there is evidence that variations within a selection of the residues are involved in binding with the S-protein. The majority of these variants display a similar binding affinity for SARS-CoV-2; however, the alleles rs73635825 and rs143936283 demonstrate noticeable variations in their intermolecular interactions with the S-protein. These variations adversely affect the stability of the encoded protein complex, resulting in a low binding affinity with the SARS-CoV-2 S-protein. This could possibly cause a change in the overall negativity of the ACE2 surface deep channel, suggesting the possibility of a natural, intrinsic resistance of a certain degree against SARS-CoV-2 [25]. It is also feasible that under new selection pressures, triggered by the recent SARS-CoV-2 pandemic, these variants may undergo positive selection.

We have performed genomic analysis of the hACE2 receptors to assess and compare the amino acids at 30 crucial variable binding sites of the ACE2 receptors based on the analysis of 70 different species. No polymorphisms were detected within *Homo sapiens* populations, indicating that binding sites may be specific to taxon groups. *Homo sapiens* were found to possess the same amino acids as Neanderthals and Denisovans at each corresponding binding site. Conservation of these ACE2 binding sites over time may have provided a survival benefit, perhaps due to the essential role of ACE2 in cardiovascular physiology [26]. However, only these 30 crucial binding sites were analysed, therefore it remains unknown if there are variations elsewhere in the ACE2 protein that alter the susceptibility of humans to SARS-CoV-2 entry.

Intriguingly, the ACE2 receptor in children demonstrates a reduced binding affinity for the S-protein and exhibits an alternate tissue distribution to adults, which reduces viral entry. The ACE2 receptor expression and affinity increases within the respiratory epithelium with age but has not yet proven to increase the infection susceptibility [27]. However, there is a reduced abundance of receptors in the elderly resulting in the accumulation of inflammatory angiotensin 2, which may contribute to the organ damage observed in COVID-19 patients, although contrasting studies showed that ACE2 prevented COVID-associated lung injury in mice [28]. Therefore, it appears that reduced ACE2 expression in the nasal epithelia of children prevents the initial entry of the virus, whereas reduced ACE2 abundance in the lungs with old age actually facilitates injury. Furthermore, high plasma ACE2 levels may potentially deactivate the virus [29].

### 2.2. Non-Human Primates

Numerous non-human primates, ranging from our closest ancestors the Great Apes to our more distant relatives the Catarrhine (Old-World) Monkeys, are susceptible to infection with SARS-CoV-2 and are likely to develop mild respiratory symptoms similar to those observed in humans with mild COVID-19. This is anticipated due to the high degree of ACE2 conservation across most non-human primate species. Firstly, the regions of ACE2 which interact with the S-protein of SARS-CoV-2, namely residues 30–41, 82–84, and 353–357 and contain five critical binding positions, are completely homologous between humans and most non-human primates [30]. Furthermore, Melin et al. [31] have discovered that the twelve critical binding residues of hACE2 were invariant in all Great Apes and Old-World Monkeys. In fact, all 21 potential binding residues identified were identical in humans, Great Apes and Old-World Monkeys [31]. In contrast, Platyrrhine (New-World) Monkeys present greater variance in three residues of ACE2 [31], and two of these, H41Y and E42Q, show strong evidence of having impactful changes on the binding affinity of the S-protein to ACE2 by approximately 400-fold [31]. Selected conserved residues within ACE2 form salt bridges and variation within these regions is also suggested to be a factor involved in the decreased susceptibility of New-World Monkeys to SARS-CoV-2. Further findings confirm the susceptibility of Great Apes to infection with SARS-CoV-2 as their ACE2 shares the same amino acids at 30 conserved sites with hACE2 [32]. Collectively, these studies strongly suggest that all Great Apes and Old-World Monkeys are susceptible to infection with SARS-CoV-2, and this is consistent with infection model studies [33,34,35,36,37,38].

Although susceptibility is undoubtable, the severity of the disease caused by SARS-CoV-2 in non-human primates is less clear. Infections have been established in non-human primates in infection model experiments, and there have also been reports of small outbreaks of mild respiratory disease caused by SARS-CoV-2 in multiple zoos across the world [39]. Unlike human patients, fever appears to be a far less common symptom in monkeys infected with SARS-CoV-2 [39]. Furthermore, there is also evidence that some infected primates are asymptomatic, which could pose problems for controlling transmission between primates within enclosures [39].

Our sequence alignment analyses confirmed existing findings that humans, Great Apes, and Old-World Monkeys share the same 12 critical binding residues in ACE2 and that ACE2 is overall highly conserved across these species, indicating that all of the aforementioned primates will display a certain degree of susceptibility to SARS-CoV-2 infection [31,32,36]. New World Monkeys show less homology in their ACE2 compared to other primates, consistent with existing studies [30]. Following these observations, Great Apes and African monkeys would be the most suitable primates to be used as models to study the vaccine efficacy against SARS-CoV-2, and if infected with SARS-CoV-2 may pose a significant risk of animal-to-human transmission.

### 2.3. Bat

Since the SARS and MERS outbreaks in 2002 and 2012, respectively; studies have suggested a link between bats and coronaviruses, specifically *Rhinolophidae* bats, which have been observed to harbour SARS-related coronaviruses [40,41]. Different coronaviruses have been identified as originating from a variety of bat species including; *Rhinolophus sinicus*, *Rhinolophus ferrumequinum*, *Miniopterus magnate*, *Pipistrellus abramus*, *Pipistrellus pipistrellus*, *Tylonycteris pachypus*, *Myotis ricketti*, and *Scotophilus kuhlii* [40]. In particular, two novel human coronaviruses were identified in the horseshoe bat species *Rhinolophus ferrumequinum* and *Rhinolophus sinicus* [42]. Uniquely, infected bats harbouring coronaviruses do not show any overt clinical signs of the disease, resulting in the assumption that bats are most likely the ancestral reservoirs for several viruses, including coronaviruses [3]. A recent study revealed that the whole genome sequence of SARS-CoV-2 was closely related (96.2% similarity) to a bat coronavirus named RaTG13, detected in *Rhinolophus affinis* (horseshoe bats) located in the Yunnan Province in China [3,43].

Bats are recognised for having a low rate of tumorigenesis and the remarkable ability to achieve a bat–virus equilibrium [44]. Studies have indicated that millions of years of adaptive evolution have shaped bats’ host immune system to develop a unique balance between antiviral defence and disease tolerance, resulting in their exceptional ability to act as an ideal host for a myriad of viruses [44]. In recent years, a link has been established between several viral outbreaks (both new and re-emerging) and spillover events from bat reservoirs, highlighting the future risk of potential spillover to human populations [44]. Detection of SARS-related CoVs in bats led to the recognition of the novel relationship between CoVs and bats [42].

As mentioned before, horseshoe bats are very likely the natural reservoir of SARS-CoV-2, given that its genome shares a high degree of conservation with the bat coronavirus (RaTG13) genome [45]. Phylogenetic analysis of the complete virus genome, S-protein and RNA-dependent RNA polymerase genes confirmed that RaTG13 is the closest known relative to SARS-CoV-2 [3]. Huang et al. [46] analysed 285 ACE2 receptor of SARS-CoV-2 variants and found mammals to be at the highest risk of infection, attributed to the low binding energy needed for S-protein interaction with ACE2 receptors. Of all the mammals they investigated, subsequent binding analysis revealed that the greater horseshoe bat (*Rhinolophus ferrumequinum*) had a relatively low binding energy (−44.47 EEU), implicating their heightened susceptibility to SARS-CoV-2. Bats that were found to possess a specific Y41H/Q42E substitution within the ACE2 receptor achieved a binding energy score of less than −47 EEU, making them more susceptible to SARS-CoV-2 infection. This is attributed to the fact that H41 in place of Y41 can no longer form a hydrogen bond to T500, lowering the van der Waals packing energy, and that substitution of Q42 with glutamic acid cannot form hydrogen bonds with G446 and Y449, disrupting the high affinity S-ACE2 interaction and hence clarifying the permissiveness of bats to SARS-CoV-2 [46].

Sequence comparison between SARS-CoV-2 and RaTG13 revealed high sequence homology in the S-protein ectodomain, with a low degree of conservation in the S-RBD. Such changes are likely to contribute to the low binding energy required for S-ACE2 binding in bats. Structural analyses of the bat virus (RaTG13) S protein identified a tyrosine substitution instead of Gln493 is unable to form a hydrogen bond with ACE2 Glu35. In addition, Glu484 substitution with threonine cannot form an intramolecular salt bridge with ACE2 Lys31 and a Tyr498 instead of Gln498 leading to inability to form a hydrogen bond with ACE2 Tyr41 [47].

The ACE2 protein contains 24 essential amino acid sites that facilitate stabilisation of the interaction occurring between the RBD within S1 of SARS-CoV-2 and the protease domain (PD) of ACE2 [48]. Frank et al. [49] have recently identified that among 24 essential ACE2 amino acid sites, referred to by their position in human ACE2, there were 132 unique combinations of amino acids within the analysed 207 different bat ACE2 sequences. This work also identified a minimum of 82 unique amino acid sequences across a subset of 7 amino acids known as virus-contacting residues which are vital for salt bridge maintenance [49]. Additionally, analysis of bat ACE2 sequences revealed that many of the key 24 ACE2 binding amino acid sites, including Phe^28^ and Arg^357^, were identified to have 10 or more potential amino acids [49]. A total of 22 sites in bat ACE2 also possess more than a single amino acid, and the degree of diversity among these sites was found to be high in bats [49]. Importantly, these findings indicate that bats are driving the signal of mammalian selection and adaptation to SARS-CoV-2 via the observed variability in bat ACE2 variable sites [50]. Further evidence to support this includes the identification of 19 accelerated residues within ACE2, which interact with SARS-CoV-2, including the positively selected codons Q24 and H34. Although it is worth considering that Q24 in humans apparently does not exhibit polymorphism [51]. Notably, studies have examined a total of six binding residues known to interact with SARS-CoV-2 in Chiroptera, Rodentia, and Carnivora and have identified that five of these residues show positive acceleration and the codon G345 is accelerated in all three lineages [51].

ACE2 sequences in the *Rhinolophidae* family are known to exhibit a high degree of polymorphism. When utilising *R. sinicus* as a model, Guo et al. [50] reported that ACE2 sequence homology lays between 98–100% at the species level, and 80–81% amino acid sequence homology was observed compared to hACE2. Significant variations in sequences have been observed in the N-terminal region of *R. sinicus* ACE2, whereby nonsynonymous single-nucleotide polymorphisms (SNPs) identified eight variable residues (24, 27, 31, 34, 35, 38, 41, and 42) and the combination of these eight residues result in eight distinct alleles (RIESEDYK, LIEFENYQ, RTESENYQ, RIKSEDYQ, QIKSEDYQ, RMTSEDYQ, EMKTKDHQ, and EIKTKDHQ or alleles 1–8) [50]. Upon statistical analyses, a total of 19 amino acid residues within ACE2 were found to be important for interaction with SARS-CoV-2, and these resides are undergoing positive selection compared to other residues in ACE2 [48]. The plausibility that the binding region of ACE2 is evolving is a very feasible prospect but requires further investigations. More studies were conducted to demonstrate similar findings and investigated the selection pressure on aminopeptidase N (ANPEP) in response to coronaviruses in other mammals [52].

### 2.4. Minks

European (*Mustela lutreola*) and American (*Neovison vison*) minks have been proven to be susceptible to SARS-CoV-2 infection, resulting in the implementation of mass culling in several countries to prevent transmission of the virus between both mink–mink and mink–human populations. Case studies from 2020 in Danish and Dutch farms revealed that minks can be infected with SARS-CoV-2 and transmit the virus back to humans [53]. In April 2020, Dutch authorities confirmed that a number of employees on mink farms were infected with SARS-CoV-2 via transmission from the minks. The ease of transmission from minks to humans is likely as minks appear to possess a similar ACE2 receptor to humans, alongside the presence of newly emerged SARS-CoV-2 variants that can efficiently bind to mink ACE2 [53].

The RBD of SARS-CoV-2 S-protein interacts with the human ACE2 receptor [54]. The sequence analysis of the hACE2 (Protein ID—BAB40370.1 (full length sequence)) and mink ACE2 (*Neovison vison* Protein ID—CCP86723.1 (partial sequence); *Mustela lutreola* Protein ID—QNC68911.1 (full length sequence)) revealed that the European mink ACE2 protein is 805 aa similar to hACE2, while the American mink ACE2 protein (partial sequence) is only 471 amino acids long. Investigation of 22 of the documented residues of hACE2 known to interact with SARS-CoV-2 were aligned with those of both European and American mink ACE2 sequences. It was evident that multiple ACE2 residues differed between minks and humans but importantly were still found to interact with the same residues on the S -protein of SARS-CoV-2 [54,55,56]. The N-terminus of the human ACE2 comprises residues from 19 to 83, and a central sequence region comprises residues from 324 to 393 [54,56]. Within the N terminus, the European mink shows nine differences in the amino acids that are important for interactions between the ACE2 and SARS-CoV-2, whereas the American mink only displays three differences. The majority of ACE2-S protein interactions appear the same across all three ACE2 sequences, which are important in mink susceptibly to SARS-CoV-2. Sequence alignment of ACE2 proteins of multiple species concluded that five amino acid residues, 353-KGDFR-357, are present in the ACE2 of most examined species [54], highlighting the importance of this key area of sequence for further investigation when considering the susceptibility of different species to SARS-CoV-2 infection. Sequence analysis further identified that mink carry a different residue at position 354 compared to humans (G354), whereby the European mink and American mink possesses R354 and H354 respectively, these alternative residues could influence the binding of SARS-CoV-2 to the ACE2 receptor. Structural remodelling carried out by Hayashi et al. [54] suggested that the H354 substitution in the American mink can increase the binding affinity of ACE2 to SARS-CoV-2 [54].

**Figure 3:**
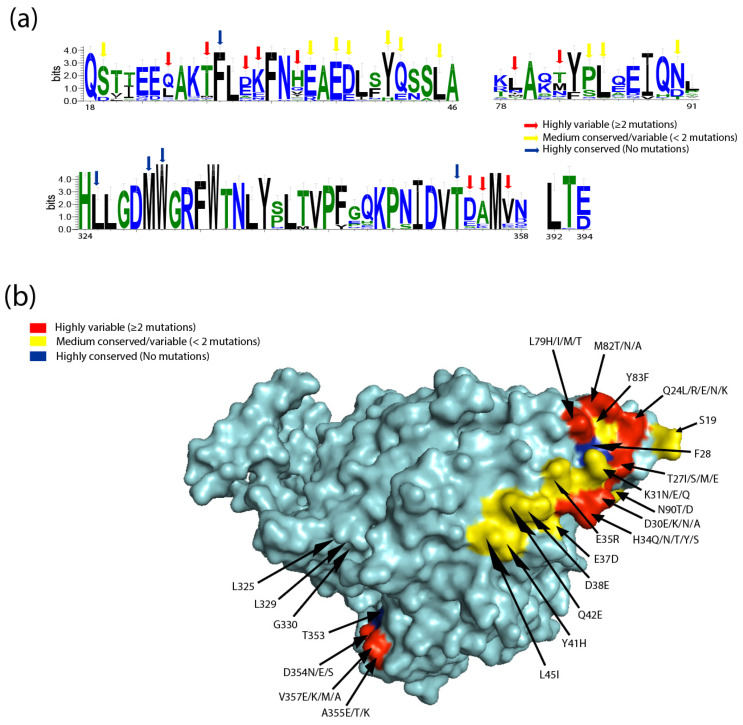
The binding sites for SARS-CoV-2 on ACE2 show a high degree of variation. (**a**) WebLogo graphs illustrating the amino acid divergence between mammalian and the ACE2 sequences of different species. The vertical height of the amino acid (aa 18–46, 78–91, 324–358, and 392–394) represents its predominance at each location in the polypeptide (aa 18–46, 78–91, 324–358, and 392–394). WebLogo (University of California, Berkeley, USA) [57] plots summarizing the amino acid divergence within the mammalian and ACE2 sequences of the different species included in this study. (**b**) Conservation of mammalian ACE2 amino acid residues, estimated from site-specific evolutionary rates, mapped onto the surface of the ACE2 ectodomain, and coloured: red (highly variable (≥2 mutations)), yellow (medium conserved/variable (<2 mutations)), and blue (highly conserved (No mutations)). Inset depicts the SARS-CoV-2 binding region of ACE2, with residues that contact the SARS-CoV-2 RBD highlighted.

The interaction of SARS-CoV-2 S1 with the hACE2 protein is associated with five amino acid residues located between positions 353 and 357 [54]. Studies investigating the interaction of the RBD of the S-protein and the American mink ACE2 have suggested that the three-dimensional structure of the binding regions complement each other, proposing that American mink are susceptible to, and can be infected by, SARS-CoV-2 [54]. It is crucial to mention that out of all tested animals, only minks are highly susceptible to SARS-CoV-2. American mink showed a slight variation in the five amino acid residues: 353-KHDFR-357, which are 353-KRDFR-357 in European mink. Investigation of the structurally complex data revealed that mink ACE2 amino acid H364 lies in close proximity to the loop structures of the RBD that aid in concentrating hydrogen bonds between residues [54]. In addition, structural remodelling revealed that the G amino acid substitution with H in five key amino acid residues in the surface motif of mink ACE2 was efficient in increasing the binding affinity of SARS-CoV-2. Overall, the virus–receptor engagement is dominated largely by polar contacts mediated by hydrophilic residues. A single substitution at G354H was sufficient to strongly conserve these interactions in minks [53]. Similar to the substitution already observed in American minks, the G354R substitution in the ACE2 of European minks should also be investigated as it might potentially influence the binding affinity to SARS-CoV-2. A comprehensive and comparative structural analyses of ACE2 receptors showed residues with variable similarity and divergence (Figure 3a,b). Alignment of the hACE2 with other ACE2 sequences of different species using the WebLogo tool revealed a number of highly variable residues within the overlapping SARS-CoV-2 binding sites, including Q24, D30, K31, H34, L79, and G354 (Figure 3a). In addition, examination of amino acid conservation at the SARS-CoV-2 binding sites on the surface of ACE2 protein sequences revealed a high degree of variation, suggesting that SARS-CoV-2 receptor binding may vary between potential hosts (Figure 3b).

Several variants of SARS-CoV-2 have emerged as a result of mink infections. One variant, known as Cluster 5, was detected in 12 people in Denmark [58]. Cluster 5 contains five different mutations; Y453F, 69-70 delHV, 1692V, M1229I, and S1147L. There is particular interest around the Y453F mutation as it encodes a mutation from Tyrosine to Phenylalanine at position 453 in the RBD of the S-protein, which seems to affect how this SARS-CoV-2 variant binds to both human and mink ACE2 [58]. The Y453F interacts with H34 in human ACE2, compared to Y34 in European mink ACE2, resulting in a better binding affinity between Y453F and mink ACE2, compared to human ACE2. However, this does not mean that Y453F does not have a high affinity for human ACE2. Deep mutational scanning indicated that variants carrying the Y453F mutation still have a higher affinity for hACE2 than wild-type SARS-CoV-2, which could lead to sustained transmission of the variant within the human population [55]. However, it has been suggested that the Y453F mutation does not bind as strongly to human ACE2, so further research is required to determine its binding affinity [54]. These variants have occurred due to the selection pressure of mink ACE2 compared to human ACE2. For example, the protein at position G354 in the human sequence is different from both proteins in the European mink R354 and H36 in the American mink ACE2. The interaction of SARS-CoV-2 at this position of European mink ACE2 led to N501T mutation, to better interact with the European mink ACE2 receptor [55].

### 2.5. Rabbit

Rabbits have been identified to exhibit a strong and consistent ACE2 binding to the S1 subunit of the S protein of SARS-CoV-2, thus implying efficient viral entry [59]. In a recent study, New Zealand White rabbits (*Oryctolagus cuniculus*) were inoculated with SARS-CoV-2 and observed to excrete infectious virus from their airways [60], highlighting their potential for both infection and transmission. However, it should be acknowledged that these results might not reflect the real-world viral behaviour as there are unknown factors that remain uncontrolled.

### 2.6. Rodents

#### 2.6.1. Hamster

There are several reports suggesting that hamsters have varying susceptibility to SARS-CoV-2. Pathogenesis and transmission have been reported in Golden hamsters (*Mesocricetus auratus*), whereby aerosol transmission and direct contact was proven to cause infection in other hamsters [61]. In addition, Golden hamsters seem to be very susceptible to infection, with a study determining the infectious dose (ID_50_) to be only five infectious particles [62], further indicating the ease of infection and transmission between individuals. Another animal model, the Roborovski Dwarf hamster (*Phodopus roborovskii*) has been described as highly susceptible to SARS-CoV-2 and even exhibits severe lung damage, reflective of that observed in human patients [63].

To gain a better understanding of rodent susceptibility to SARS-CoV-2, rodent ACE2 sequences were aligned with hACE2 using the programme Needle. Results show that Golden hamsters had a 91.7% similarity, Roborovski Dwarf hamsters had a similarity of 91.6%, and the New Zealand White rabbit had a 92.8% similarity to hACE2. It is assumed that out of the studied rodents, the New Zealand White rabbit holds the closest relation to the hACE2 receptor. Similarities between rodent ACE2 sequences are 85.47% and 86.46% in the New Zealand White rabbit vs. Dwarf and Golden hamster, respectively, and similarity between both hamster species was 96.89%. In order to investigate the specific differences between the hACE2 and rodent ACE2 further, it should be determined if differences in sequence will affect the contact residues within the ACE2-S interface. Important key residues of hACE2 include: Q24, T27, F28, D30, K31, H34, E35, E37, D38, Y41, Q42, L79, M82, Y83, N330, K353, G354, D355, R357, and R393 [56]. The ACE2 sequence of the Golden hamster demonstrated two major changes in these key residues, whereby H34 had been replaced by Q34 and M82 to N82. The Roborovski Dwarf hamster ACE2 sequence had four major changes, whereby H34 has been replaced by Q34, M82 to N82, G354 to E354, and Q325 to K325. Finally, the New Zealand White rabbit exhibited four major changes; Q24 to L24, D30 to E30, H34 to Q34, and M82 to T82. Intriguingly, the New Zealand White rabbit presented changes to amino acid residues, which directly act on hydrogen bonds and salt bridges at the ACE2-S interface, and the Roborovski Dwarf hamster also showed amino acid changes directly involved in hydrogen bonds. As a result, these amino acid changes could either strengthen or weaken the bonds between the ACE2 receptor and the S-protein and thereby affect host susceptibility.

#### 2.6.2. Mouse and Rat

It has been demonstrated that wild-type mouse models are poorly infected with SARS-CoV-2 and only show weak transmission due to SARS-CoV-2 remaining unable to bind to the mouse ACE2 receptor [3]. Also, the rat ACE2 receptor cannot be bound by to SARS-CoV-2 S-protein [64]. It can be determined that mouse and rat populations are unlikely to be involved in the transmission of SARS-CoV-2 due to differences in the basic structure of their ACE2 receptors. Despite their inability to be infected with SARS-CoV-2, these rodents can still be utilised in SARS-CoV-2 experimental studies. For example, the use of transgenic mice expressing hACE2 has been well described, allowing the infection and replication of SARS-CoV-2 in lungs [65]. Such studies identified that these transgenic mice cannot develop severe SARS-CoV-2 infection that would result in pneumonia or fatality [65]. The use of hACE2 transgenic mice also proved to be useful in confirming that human-to-human transmission of SARS-CoV-2 largely occurs due to close contact between individuals [66].

The structures of the SARS-CoV-2 RBD, ACE2 N-terminal helix, and their interactions have now been well characterised [56]. Based on these structural analyses, there are eight amino acid differences in mouse and rat ACE2 compared to human N-terminal regions of the ACE2 receptor. In *Mus musculus* ACE2, these residues were N24, N30, N31, Q34, T79, S82, F83, and H353 while in *Rattus norvergicus*, these residues were K24, N30, H34, I79, N82, F83, and H353. Therefore, these differences were not identical in mouse and rat ACE2. One key difference in residues between human and rodent ACE2 is H353, which is shared in both mouse and rat ACE2. It has been previously observed that a H353K substitution (to match that of hACE2) can allow SARS-CoV-2 S-protein binding in rodents [67]. Therefore, it can be concluded that this mismatch is a likely candidate for preventing SARS-CoV-2 S-protein binding to mouse and rat ACE2. Furthermore, hACE2 K353 interacts with Q498 of the SARS-CoV-2 RBD via hydrogen bonding. Hence, the H353 residue is likely the cause of reduced interaction in mouse and rat ACE2 [67].

### 2.7. Pets and Zoo Animals

#### 2.7.1. Cats and Ferrets

SARS-CoV-2 is able to replicate in the nose and throat of cats, whilst causing pathology in the upper respiratory tract that is associated with inflammation. Additionally, airborne transmission has been documented between cats. Similar to cats, SARS-CoV-2 can also be found in the upper respiratory tracts of ferrets; however, only a poor transmission was documented between individuals [67]. It is hence conceivable that cats are more susceptible to SARS-CoV-2 than ferrets and it would be expected that cats possess a more similar ACE2 sequence to hACE2 than ferrets do. Analysis of the ACE2 sequences of these animals justified the differential susceptibility between cats and ferrets for SARS-CoV-2. The amino acid sequence of hACE2 is 85.2% similar to that of cat ACE2, compared to only an 82.6% similarity with ferret ACE2. Furthermore, out of the 19 key residues required for S-protein binding to ACE2, four and five were altered in cats and ferrets, respectively. These results suggested that cats are more susceptible to SARS-CoV-2 infection than ferrets [68]. The KGDFR region of hACE2 receptors appeared to be conserved between humans and several mammals [54], including cats, yet this region is not the same in ferrets (KRDFR). This further suggests that ferrets show a diminished susceptibility to SARS-CoV-2 compared to humans and cats. Therefore, the poor transmission between ferrets is likely associated with this reduction in binding affinity.

#### 2.7.2. Zoo Animals: Big Cats

One of the leading factors mediating the transmission of SARS-CoV-2 from humans to animals is the human activity, which results in ramifications for animal and human health management alongside wildlife conservation. With the viral origin and causative agent of the COVID-19 pandemic having arisen from a natural animal reservoir, the health implications to humans and other animals remains tremendously high [69]. The first reported infection of a tiger with SARS-CoV-2 occurred at the Bronx Zoo in New York, whereby the tiger was infected with the virus via transmission from its handler. Subsequent to this, it became evident that the virus can spread from humans not only to small mammals but also to large wild animals. The Bronx Zoo later reported the infection of three more tigers and three lions with SARS-CoV-2 in the days following the first infection [70]. It is interesting to note that infection of these big cats at the Bronx Zoo occurred at a time when testing for SARS-CoV-2 remained limited and little information was available on the dynamics of SARS-CoV-2 viral shedding [71].

At present, it is plausible to assume that tigers and lions can re-infect humans due to close interactions and a very strong evolutionary association between SARS-CoV-2 in humans and big cat groups [70]. McAloose et al. [70] have determined that the genotypes of the SARS-CoV-2 strains affecting lions and tigers are actually distinct from each other and the genetic architecture of the virus isolated from one of the tigers in the Bronx Zoo was identical to the asymptomatic animal handler from where the virus was transmitted. Furthermore, the isolated virus from the tiger and handler was the same strain that infected the larger human population in the city of New York. Studies have shown that five key amino acids that facilitate the interaction between the S-protein of SARS-CoV-2 and the ACE2 receptor are highly conserved in humans, companion felines, and wild felines such as tigers [68,72]. The KGDFR region appears to be of particular importance for SARS-CoV-2 binding to ACE2, and by extension, susceptibility of the species to the disease. Based upon the reports of tigers being infected with SARS-CoV-2, it is of no surprise that this region of the receptor sequence is conserved in tigers as well as leopards that also demonstrated conservation of the KDGFR region of the ACE2 receptor. Although there are no reports of leopards’ susceptibility to SARS-CoV-2, this conserved region would suggest that they are just as susceptible as tigers, and transmission between individuals is conceivable.

#### 2.7.3. Dogs and Civets

Domestic animals such as cats and dogs that are in regular close contact with humans can be susceptible to SARS-CoV-2 infection. Analysis of SARS-CoV-2 infected beagle dogs concluded that replication of SARS-CoV-2 was poor, and no viral particles were detected in any major organs or tissues. Furthermore, the transmission of SARS-CoV-2 in beagle dogs was also poor, indicating that dogs possess a low susceptibility to SARS-CoV-2 infection [73]. The ACE2 receptor in dogs has a higher free binding energy (−40.7 KJ·mol^−1^) than humans (−50.1 KJ·mol^−1^), signifying that the binding ability of the dog ACE2 receptor to the S-protein on SARS-CoV-2 is weaker than that of hACE2 [74]. This reduction in affinity may be due to mutations in residues that are critical for binding to the RBD of the S-protein. The dog ACE2 amino acid sequence is 83.5% similar to the hACE2, possessing five substitutions at Q24L, D30E, D38E, M82T, and H34Y. The Q24L and M82L mutations are disruptive in the binding of ACE2 to the S-protein, and the H34Y mutation reduces the binding affinity of ACE2 to the S-protein significantly by affecting the hydrogen bond at Y453 of RBD of the S-protein [75]. It is important to note that human–dog transmission still remains possible, as evidence has proven a case of SARS-CoV-2 infection in a Pomeranian dog in Hong Kong [76]. However, it remains currently unknown if transmission can spillover from dog to human.

Palm civets (*Paguma larvata*) were identified and confirmed as the main intermediate host for the previous coronavirus outbreak, caused by SARS-CoV. Unsurprisingly, research has determined that palm civets are also susceptible for infection by SARS-CoV-2. The palm civet ACE2 receptor displays a binding affinity to the S-protein (−37.1641 KJ·mol^−1^) based on the free binding energy analysis compared to dogs (37.389 KJ·mol^−1^) and humans (−37.389 KJ·mol^−1^) [74]. However, the *Paguma larvata* ACE2 amino acid sequence only shows 83.48% similarity to the hACE2 receptor sequence and differs in six of the key amino acid residues required for S-protein binding [77].

As previously mentioned, cats and dogs are both susceptible to SARS-CoV-2, as evidenced via human-to-animal transmission [68,73]. Despite no evidence of spillback events’ from pets to human, it may become necessary for vaccination of such animals to avoid the potential spread and evolution of the virus. As cats and dogs are host reservoirs, the virus retains the potential to evolve and develop animal-specific strains, which may in turn be transmitted to humans, leading to a more virulent strain of SARS-CoV-2.

### 2.8. Livestock

The types of animals used as livestock vary globally, but livestock are typically always in close proximity with humans. If these animals were susceptible to infection with SARS-CoV-2 and are able to transmit the virus, this could pose a huge threat to food security. Viruses have previously spread via the clinical infection of livestock and subsequently via their meat and dairy products, such as tick-borne encephalitis [78]. However, this is rare, and food products are usually contaminated through the environment, for example by workers handling the products. In addition, the virus would have to be extremely robust to survive the chilling, freezing, and high heat cooking procedures involved in food processing. During the period from March to October in 2020, China reported that SARS-CoV-2 had been detected on the outer package of imported Brazilian beef and South American white shrimps on several occasions [79]. In August alone, 14 batches of food were not allowed to enter the country due to the detection of animal diseases and were returned or destroyed at the port according to law [79]. However, these positive results have only been detected on the outer packaging, and there is no evidence that the virus can survive in cells following food processing.

Computer modelling and analysis indicates that ACE2 proteins of different livestock show a high similarity to hACE2 [80]. Notably, HeLa cells expressing ACE2 of cattle, sheep, and pigs have all been proven to be successfully infected with SARS-CoV-2 [81]. Respiratory ex vivo organ cultures inoculated with SARS-CoV-2 demonstrated that cows and sheep can sustain viral replication, whilst pigs are unable to withhold infection with SARS-CoV-2. This is likely because cows and sheep express more ACE2 in the respiratory tract than pigs [82]. In another study, cattle were experimentally inoculated with SARS-CoV-2 and observed viral replication showed that there is no viral transmission to other naïve cattle [83]. Similar studies in pigs failed to detect viral replication, viral shedding, or seroconversion [84]. One explanation for these results is that ACE2 of cattle, sheep, and pigs may be able to successfully bind to SARS-CoV-2, but the virus cannot be completely maintained and replicated in cells to the extent that it can be transmitted. It is also likely that pigs may not express enough ACE2 to support the viral entry, which is endorsed by the observation that there are very few ACE2-expressing target cells in pigs and goats [85]. Alternative explanations have been considered and systematically eliminated following preliminary analyses. For example, the use of bovine respiratory vaccine since 2010 has significantly reduced the cases of bovine respiratory coronavirus [86]. Bovine respiratory coronavirus and SARS-CoV-2 are both betacoronaviruses with high similarity, so it was theorised that infection with one may confer resistance to the other. Two challenges with this explanation are that most wild species of cattle were not vaccinated, but still cannot be infected. In addition, Ulrich et al. [83] have stated that the presence of a pre-existing coronavirus did not protect the host from infection with another betacoronavirus.

Limited studies have been performed on other livestock species. Alpacas have shown sero-reactivity following inoculation with SARS-CoV-2, but it is unclear if they could naturally be infected with the virus [87]. Camels, which are another important livestock species globally, have thus far not been linked to SARS-CoV-2, despite them being the intermediate host for the previous MERS outbreak [88]. Overall, there have been no case studies of livestock species becoming naturally infected with SARS-CoV-2 and no indication of them being able to transmit the virus. However, there would be a huge threat if a new strain of the virus was able to infect these animals, and it is hence imperative that there is continued surveillance for livestock species.

Investigation into the ACE2 sequence of livestock species and comparison of sequence similarity to hACE2 has been undertaken. Investigation of all livestock species demonstrated variations in amino acid structure sequence from that of the hACE2 receptor. Two substitutions were observed in goats and sheep, whereby a methionine to threonine substitution was identified as semi-conservative with a potential impact on ACE2 structure, feasibly as a result of an increased capacity to form hydrogen bonds. The same may apply to cows because, even though they exhibited three substitutions, only the methionine to threonine substitution would appear to have a structural impact, again through changes to the ability to form side chain hydrogen bonds. Pigs, camels, and alpacas all exhibit variation from hACE2 at four amino acid sites. Substitutions in pigs were associated with either complete loss of the ability to form H-bond side chains at that site, as well as changes in hydrophobicity, side chain flexibility, or from nonpolar to polar (and vice versa) amino acids. In pigs, the H34L substitution may be of particular significance, leading to a loss of ability for ionic bonds, H-bonds, and aromatic stacking. Similarly, in camels, one of these substitutions is associated with a complete loss of H-bond potential, whilst others are associated with a gain from 0 to 3 side chain H-bonds. Substitutions in alpacas are principally associated with changes in H-bond side chain formation and side chain flexibility. These modulations are potentially sufficient to adapt ACE2 structure and change binding affinity with the SARS-CoV-2 S-protein. Overall, it can be determined that there is no direct evidence that livestock currently possess the potential to act as the intermediate host for SARS-CoV-2. However, observation should be maintained to prevent any future risks that could arise from mutations in the S-protein of SARS-CoV-2 variants.

### 2.9. Bird

Alpha- and Betacoronaviruses are known to have the capacity to infect mammals but are typically unable to infect birds, whereas Delta- and Gammacoronaviruses are usually able to infect birds and subsequently cause disease [89]. However, several studies have aimed to determine whether this still holds true for SARS-CoV-2. Suarez et al. [90] have investigated the susceptibility of five species of poultry, including chickens (*Gallus gallus*), turkeys (*Melegris gallopavo*), and ducks (*Anas platyrhinchos*). After inoculating the birds with SARS-CoV-2, they were monitored for virus presence for 7 days post-infection and for antibodies 14 days post-infection. Results showed that SARS-CoV-2 could not replicate within the birds and that no antibodies were generated in response to the inoculation. Studies by Schlottau et al. [84] have found similar results that confirmed no viral shedding, tissue damage, or antibodies occurred in chickens and turkeys. Interestingly, both studies also concluded that embryonated chicken eggs, often used to grow influenza A and some of the avian coronaviruses were unable to support SARS-CoV-2 replication [84].

Alexander et al. [80] have identified 24 residues important in hACE2-S protein binding, and within this group of amino acids seven residues were identified (24, 30, 31, 34, 79, 83, 329) that correlated with increased susceptibility of animals to SARS-CoV-2 infection. Further analysis indicated that glycosylation at amino acids 90 and 322 were important in determining the susceptibility of animals to SARS-CoV-2 infection [80]. A total of 20 out of these key ACE2 residues have also been identified by Luan et al. [91] as having important roles within these binding interactions. When comparing the ACE2 sequence of birds to hACE2, residue 24 in humans, ducks, and vultures all appear to have a polar glutamine, whereas in wild turkeys and chickens they have negative glutamate residues. Additionally, in hACE2, Asp30 interacts with the S-protein of SARS-CoV-2 at a site adjacent to the RBD and is important to form a salt bridge. However, in chickens, ducks, and turkeys this has been replaced with aliphatic alanine residues which cannot form a salt bridge [56]. Moreover, positively charged Lys31 in the hACE2 receptor is known to be important for binding the S-protein, but in all analysed five bird species, this amino acid has been substituted to a negatively charged glutamate. Further changes include that the positively charged His34 in humans is not conserved in birds, which alternatively possess either aliphatic valine residues or a polar proline. These findings are significant because Asp30, Lys31, and His34 are thought to interact with Leu455 of the S-protein, therefore any alterations to the charges at these residues may impact the ability of the ACE2 receptor to interact with SARS-CoV-2 [56].

Other key substitutions between human and bird ACE2 residues include position 79 in hACE2, which is a positively charged lysine, whilst all bird species have polar asparagine residues. In hACE2 Tyr83, a nonpolar aromatic tyrosine residue binds with Asp83 in the RBD of the S-protein through hydrogen bonding. However, in five analysed birds, this is substituted with an aromatic phenylalanine residue [92]. Analysis showed that there is also no conservation observed at position 329 between hACE2 and the birds ACE2 receptor. Aside from the specific sequence in ACE2, glycosylation of Asn90 and Asn322 is thought to facilitate ACE2-S binding; however, no glycosylation is observed in these residues in birds [92]. In humans, Gly354 is thought to be associated with susceptibility to infection, but in ducks and chickens it is altered [90,91].

There is a relatively high conservation of ACE2 receptors, with 65% identity of the key residues involved in binding the S-protein of SARS-CoV-2 across five different bird species. Discrepancies in bird ACE2 conservation include duck and vultures possessing a negative Glu24 residue, whilst chickens and turkeys have a polar Gln24 residue. Despite having a different amino acid at this position, these residues are similar in size and, in some cases, polar residues can substitute for charged residues with minimal impact on its function. Additionally, ducks have an aliphatic Met27, chicken and turkeys have a polar threonine at this position, and vultures have an aliphatic isoleucine. These residue alterations exhibit major changes, as two are non-polar and one is polar, which will impact on the electrostatic interactions and therefore could change the ACE2 protein conformation. Markedly, vultures are unusual in that they possess a negative Glu30, yet ducks, turkeys, and chickens all possess aliphatic alanine at this position, which is a much smaller residue lacking charged groups. Similarly, most birds have an aliphatic Val34, whilst vultures have polar proline at this position, which can influence protein conformations due to its rigid ringed structure. Position 38 of the ACE2 receptor displays similar trends, whereby most birds have a negative aspartate, but vultures have a polar asparagine, although this alteration may have less of an impact as it is similar in size and charge. Another important residue in which birds display polymorphism is residue 82. Ducks have a polar asparagine at this site, vultures have a polar serine, while turkeys and chicken possess a positive arginine. These residues significantly differ in size, which has the potential to influence ACE2 receptor-S binding, although they display similar charged properties, which may reduce its impact on the protein. Furthermore, vultures have a positive Lys322, whilst other bird species have a polar asparagine, and these residues share similar bonding properties. Most birds possess a negative Glu325, but turkeys have an aliphatic alanine. The final key polymorphic residue worth mentioning is 329, whereby ducks possess a positive lysine, chickens and turkeys have a polar threonine, and vultures have a polar asparagine. Despite sizing differences, overall the displayed properties here are quite similar and as such the binding capabilities of ACE2 receptors are unlikely to be majorly impacted by these amino acid substitutions. Overall, the majority of examined residues in avian receptors appear to be largely conserved, and where they are not, polymorphic substitutions often retain similar binding properties and are hence unlikely to affect the binding ability of ACE2 to the S-protein [80,91].

Overall, amongst the bird species studied, there is relatively little difference between the key amino acid residues in their ACE2 receptors, with turkeys and vultures markedly differing the most in their residues. However, there is a significant difference in the amino acid sequence between hACE2 and the ACE2 receptor of birds that is worth noting because there are many substitutions at the residues involved in binding the S-protein, and little to no conservation is retained of the binding properties, size, and charges of these residues. This suggests that, despite some homology within the overall sequence of the ACE2 receptor, birds are unlikely to be susceptible to SARS-CoV-2 infection as their key S binding residues are unlikely to bind as efficiently to SARS-CoV-2, although it is difficult to completely guarantee this as the amino acid sequence is not the only determining factor in ACE2-S binding [80].

### 2.10. Pangolin and Snake

#### 2.10.1. Pangolin

Pangolins (*Manis javanica*) have been previously reported as feasible intermediate hosts due to their known capability to host a betacoronavirus that shares a 90% sequence identity with SARS-CoV-2 [93,94]. In silico analysis of the S-protein of SARS-CoV-2 showed that it could bind with pangolin ACE2 similar to hACE2 [95]. These initial analyses predicted the interface between the RBD of SARS-CoV-2 S-protein, and pangolin ACE2 and showed a possible interaction. Hence, there is high potential of pangolin ACE2 to support SARS-CoV-2 entry due to the sharing of key residues within the RBD in both human and pangolin ACE2 [91].

Despite these initial results implying that pangolin ACE2 can bind to SARS-CoV-2, further studies have now disproved pangolin as the intermediate host for the virus. Initial investigations that suggested pangolin as the intermediate host were conducted solely via in silico modelling, which is not representative of how the spillover event occurred [96]. Therefore, it can be concluded that the initial data available did not fit with the proposed spillover model for the emergence of SARS-CoV-2 and the pangolin is hence unlikely to be responsible as an intermediate animal [51]. Damas et al. [51] have used comparative genomic and structural protein analysis to assess the ability of ACE2 of 410 vertebrate species, including pangolins, to serve as a receptor for SARS-CoV-2 S-protein. A total of 25 key amino acids known as binding residues for the S-protein of SARS-CoV-2 were analysed for their similarity to hACE2, and results showed that pangolin ACE2 displayed only a very low binding score [73]. Luan et al. [91] have also analysed the affinity of the S-protein of SARS-CoV-2 to bind with 20 key residues of ACE2 in many animals, including pangolin, and the sequence alignment results showed that out of these 20 residues, pangolin ACE2 only shared 14 out of them (70% similarity) [91].

Alignment of pangolin ACE2 along with hACE2 identified that pangolin ACE2 possessed seven mutated residues that are crucial for S-protein binding (Q24E, D30E, H34S, D38E, L79I, M82N and G354H). Alterations to these key binding residues are likely to contribute to the relatively low binding energy observed for pangolin ACE2 to SARS-CoV-2 S-protein. However, favorable interactions are extensively formed, including thatE38 could form two hydrogen bonds with Q498 and Y449, E30 and E24 could form a hydrogen bond with K417 and N487, respectively and S34 could form a hydrogen bond with Y453. It can be concluded that although pangolin ACE2 does facilitate binding with SARS-CoV-2, it only does so at a very low capacity, and additional research is required to identify the intermediate host of the SARS-CoV-2.

#### 2.10.2. Snake

Snakes had once been considered as the original source, or an intermediate host, of SARS-CoV-2, but this was proven not to be the case [97]. Studies aligning the key residues of the hACE2 utilized in binding with the SARS-CoV-2 S-protein have demonstrated that snakes share less than 56% similarity in their ACE2 sequences [98]. Upon investigating the potential association between SARS-CoV-2 and key residues of ACE2 receptors, several mutations were observed in the snake ACE2 compared to hACE2. For example, the amino acid H34 in h ACE2 is mutated to A60 in snake, and the residue K31 in human is replaced with Q57 in snake ACE2. This K31 residue in hACE2 is critical for binding the RBD of SARS-CoV-2, and therefore this mutant in snakes completely abolishes its ability to associate with SARS-CoV-2 [91].

The Root Mean Square Deviation (RMSD) of 14 key residues influencing the binding of ACE2 in different species to SARS-CoV-2 has been investigated by Fang et al. [98] to determine the stability of each ACE2-S complex. Assessment showed that the binding free energy of snake ACE2 when complexed with SARS-CoV-2 presents highly fluctuating properties and unstable binding modes with the lowest scoring (-33.01 kcal/mol) among the 22 studies species [98]. Additionally, upon observing the binding modes of ACE2-SARS-CoV-2 S-protein in human and snake, three regions of snake ACE2 with large structural differences were observed. Within these regions, one compact yet complicated polar interaction network was formed between hACE2 and the S-protein of SARS-CoV-2, yet these polar contacts were not present in snake ACE2, which is the main reason for the reduced ability of snake ACE2 to bind SARS-CoV-2 [98,99,100,101,102]. Further to this, Luan et al. [91] have generated structural simulations showing several key contacts of hACE2 with SARS-CoV-2 RBD and found that in snake ACE2, these contacts were completely eradicated. Overall, analyses of snake ACE2 show that this receptor is highly unlikely to be able to bind with SARS-CoV-2 S-protein due to its extremely weak binding affinity, attributed to large differences in the ACE2 sequences.

## 3. Conclusions

Structural and genetic analyses highlight the potential of different animals in harbouring SARS-CoV-2 infections and potential back and forward transmission of the virus. These insights suggest that the transmission of SARS-CoV-2, particularly its emerging variants, to previously unknown animals, poses the risk of the generation of alternate viral reservoirs. Similar to mink-associated re-emergence of infection in human, the zooanthroponotic transmission of SARS-CoV-2 is a likely event, particularly at the tail-end of the pandemic. Owing to myriads of anthropogenic factors, including climate change, deforestation, and urbanization, the emergence of future viruses is inevitable, and stopping zoonotic and zooanthroponotic spillover of viruses warrants global efforts in reducing the contact between human populations and wildlife. In addition, health should be considered as multifactorial system that can be influenced by the environment, pathogens, and human activity.
